# Spectral computed tomography angiography using a gadolinium-based contrast agent for imaging of pathologies of the aorta

**DOI:** 10.1007/s10554-024-03074-2

**Published:** 2024-02-29

**Authors:** Markus Graf, Felix G. Gassert, Alexander W. Marka, Florian T. Gassert, Sebastian Ziegelmayer, Marcus Makowski, Michael Kallmayer, Jonathan Nadjiri

**Affiliations:** 1 Department of Diagnostic and Interventional Radiology, School of Medicine & Klinikum rechts der Isar, Technical University of Munich, Ismaninger Straße 22, 81675 Munich, Germany; 2 Department of Vascular and Endovascular Surgery, School of Medicine & Klinikum rechts der Isar, Technical University of Munich, Ismaninger Straße 22, 81675 Munich, Germany; 3 Department of Interventional Radiology, School of Medicine & Klinikum rechts der Isar, Technical University of Munich, Ismaninger Straße 22, 81675 Munich, Germany

**Keywords:** Aortic aneurysm, Spectral computed tomography angiography, Gadolinium, Dual energy, Monoenergetic reconstruction

## Abstract

**Objectives:**

Especially patients with aortic aneurysms and multiple computed tomography angiographies (CTA) might show medical conditions which oppose the use of iodine-based contrast agents. CTA using monoenergetic reconstructions from dual layer CT and gadolinium (Gd-)based contrast agents might be a feasible alternative in these patients. Therefore, the purpose of this study was to evaluate the feasibility of clinical spectral CTA with a Gd-based contrast agent in patients with aortic aneurysms.

**Methods:**

Twenty-one consecutive scans in 15 patients with and without endovascular aneurysm repair showing contraindications for iodine-based contrast agents were examined using clinical routine doses (0.2 mmol/kg) of Gd-based contrast agent with spectral CT. Monoenergetic reconstructions of the spectral data set were computed.

**Results:**

There was a significant increase in the intravascular attenuation of the aorta between pre- and post-contrast images for the MonoE40 images in the thoracic and the abdominal aorta (*p* < 0.001 for both). Additionally, the ratio between pre- and post-contrast images was significantly higher in the MonoE40 images as compared to the conventional images with a factor of 6.5 ± 4.5 vs. 2.4 ± 0.5 in the thoracic aorta (*p* = 0.003) and 4.1 ± 1.8 vs. 1.9 ± 0.5 in the abdominal aorta (*p* < 0.001).

**Conclusions:**

To conclude, our study showed that Gd-CTA is a valid and reliable alternative for diagnostic imaging of the aorta for clinical applications. Monoenergetic reconstructions of computed tomography angiographies using gadolinium based contrast agents may be a useful alternative in patients with aortic aneurysms and contraindications for iodine based contrast agents.

## Key points

- Patients with aortic aneurysms may show contraindications for iodine based contrast agents but need multiple follow-up examinations.
- Monoenergetic reconstructions of computed tomography angiography using gadolinium based contrast agents is feasible in patients with aortic aneurysms.
- Dual-layer spectral computed tomography may therefore be a useful tool in patients with aortic aneurysms needing follow-up scans.

**List of Abbreviations**.

**Table:** 

Abbreviation	Definition
CTA	computed tomography angiographies
EVAR	endovascular aneurysm repair
Gd	gadolinium
HU	Hounsfield units
iDose[i4])	Iterative image reconstructions
MRI	magnetic resonance imaging
ROI	regions of interest
SPCT	spectral detector CT
MonoE	virtual monoenergetic
MonoE40	virtual monoenergetic images with 40 keV

## Introduction

There has been an increase in the incidence of aortic aneurysms both, in the thorax and the abdomen, as a result of an aging population, the introduction of screening programs, an increasing number of smokers and improved diagnostic tools [1]. Depending on the aneurysm diameter and the risk factors there are conservative therapeutic approaches such as risk factor modification and betablockers or surgical approaches through endovascular aneurysm repair (EVAR) [2, 3]. In both cases patients need regular follow up examinations through CT scans using iodine-based contrast agents [2]. Nevertheless, these patients may have contraindications to iodine-based contrast agents and not seldomly, especially in an aging population, patients present with an impaired kidney function due to application of high amounts of contrast agents during the EVAR procedure [4].

Gadolinium (Gd)-based contrast agents as used in magnetic resonance imaging (MRI) on the other hand show a more favorable safety profile. Previous studies have shown that compared to iodine-based contrast agents, Gd-based contrast agents cause substantial, albeit lower radiation attenuation [5, 6]. Additionally, severe allergic reactions are reported less often and there is less affection of kidney function when Gd-based contrast agents are applied [7]. Because of lower attenuation properties, older studies applied high doses of Gd (0.3 to 0.4 mmol/kg) to achieve adequate contrast [8]. A pilot-study by Nadjiri et al. showed that using spectral CT, Gd-based contrast agent thoracic angiography with clinical doses of Gd is technically feasible [9].

Spectral CT is capable of overcoming limitations of conventional CT by offering the possibility to extrapolate virtual monoenergetic (MonoE) data [10–12]. The novel technique to detect a multienergy spectrum is based on a dual-layer detector system [13, 14]: Lower energy photons are attenuated in the first layer so that the second layer detects a harder spectrum of the same radiation, which then results in the registration of two different energy spectra. These two spectra can be detected simultaneously and with the same amount of radiation exposure. Virtual monoenergetic images are generated from this spectral data. Using a phantom model and a dose of 0.5 mmol/kg bodyweight, Bongers et al. showed that virtual monoenergetic images with 40 keV (MonoE40), which are generated from this spectral data are especially favorable for Gd-based contrast agents [15]. Nadjiri et al. showed that using spectral CT and reconstruction of these monoenergetic images, Gd-based contrast agent angiography with clinical doses of Gd (0.2 mmol/kg bodyweight) is technically feasible in patients in a clinical off-label use [9]. Nevertheless, no study has proven the usability spectral CT imaging using Gd-based contrast agents in a well circumscribed disease or a specific application, respectively.

Therefore, the goal of this study was to evaluate feasibility of using spectral CT for angiography using Gd-based contrast agent in patients with aortic disease or post-interventional follow-up examinations after interventional aortic repair.

## Material and methods (454)

**Study poplulation** Between October 2015 and December 2021 all consecutive patients with clinical indication for angiography of the aorta but contraindications for iodine-based contrast agents were included in this retrospective study. Indication for Gd-agent-based CTA was approved as an individual healing trial in interdisciplinary consensus in each subject for clinical reasons. All patients gave written informed consent for Gd-enhanced CTA in which potential complications and “off-label-use” of Gd was discussed. Study analysis was carried out retrospectively from the registry data. This study has been approved by the local ethics committee.

21 scans in 15 patients were included in this study. The majority of patients included were male (*n* = 13). Average age at scanning time was at 72.1 ± 9.3 years (IQR: 70.0–78.8 years; range: 55–85years). Two patients showed a severe allergoid reaction after application of an iodine-based contrast agent in a previous examination and therefore declined application of iodine. The other 13 patients had a severely reduced renal function with a GFR between 30 and 45 mL/min and declined application of an iodine-based contrast agent.

### Computed tomography scans

A 64-slice single source dual-layer spectral CT system was used for imaging in all cases (IQon; Philips Healthcare). Slice thickness was set to 0.9 mm with a collimation of 0.625 mm. All images were acquired with 120 kVp using a spiral scanning technique. For each patient, 0.2 mmol/kg body weight of Gd-DOTA (Dotagraf, Berlis AG, Switzerland) was used, with a 1:1 saline dilution for bolus prolongation. For timing of the contrast phase the test-bolus technique was used: 10 mL of the prepared Gd-DOTA-saline solution mixture was applied without use of spectral data to detect the time point of peak attenuation. For the test bolus, conventional technique was used, as MonoE reconstruction is not yet available for this application. No additional delay was added. The contrast-enhanced scan was then obtained at an injection rate of 4 to 6mL/s of the above-mentioned dilution followed by a 50 mL of saline chaser bolus. Iterative image reconstructions (iDose[i4]) were obtained with 0.9 and 3 mm slice thickness using the XCA (smooth) kernel. All patients included received an enhanced scan of the thoracic or the abdominal aorta with at least about half of the abdomen / thorax additionally included in the field of view. A subset of patients also received a pre-contrast scan.

### Post processing

After acquisition, multiplanar virtual MonoE40 reconstructions were calculated from the spectral CT data set using a dedicated postprocessing software (Philips Intellispace Portal 8.0; Philips Healthcare). To evaluate changes in extrapolated attenuation, regions of interest (ROI) were placed within the thoracic and abdominal aorta in pre- and post-contrast images (ROI > 20 mm^2^). Mean attenuation values were extracted from the ROIs in both the thoracic and abdominal aorta.

### Statistical analysis

All statistical analyses were performed by F.G.G using the statistical package R version 3.2.4 (R Foundation for Statistical Computing, Vienna, Austria). Categorical variables are expressed as frequencies and percentages, continuous variables are expressed as mean ± standard deviation. The tested data have visually been evaluated for normal distribution. Two-sided t test was applied. A P-Value < 0.05 was considered statistically significant.

## Results

### Imaging and image quality

**Fig. 1:**
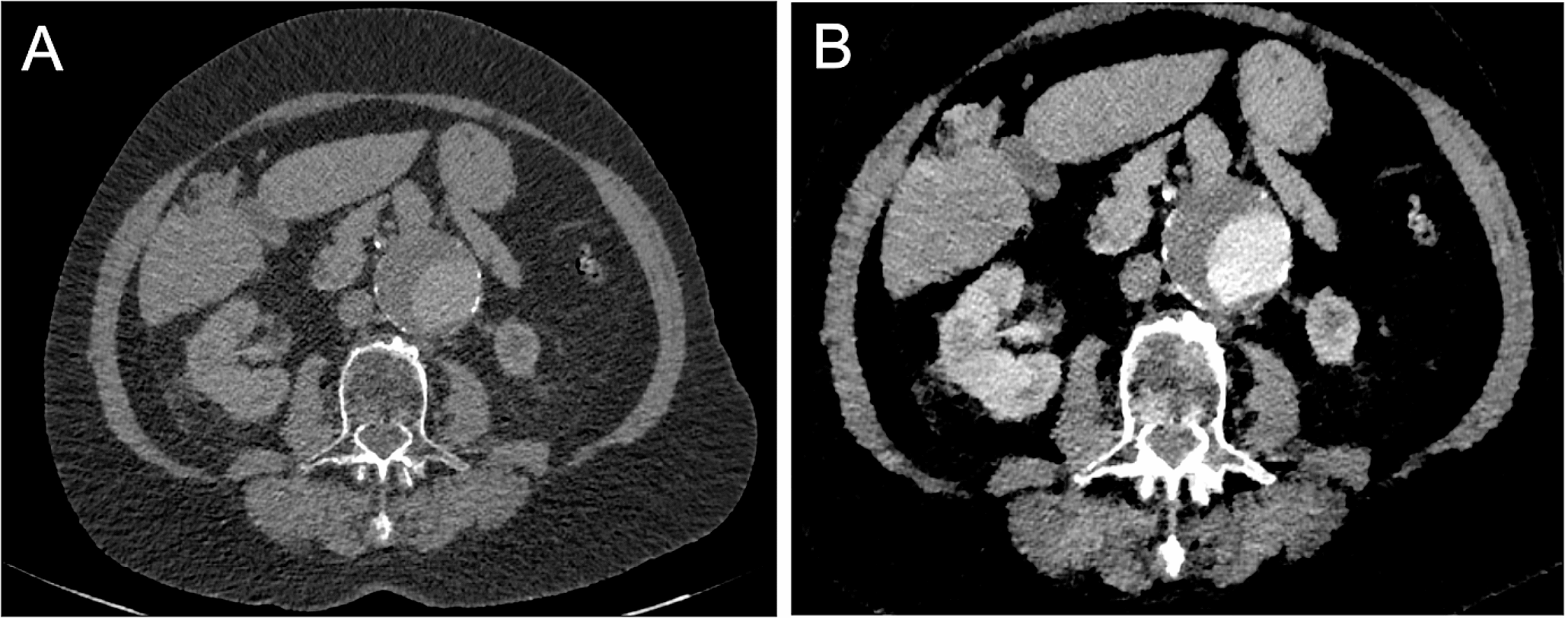
Conventional (A) and MonoE40 (B) axial reconstruction of a Gd-enhanced CT angiography of the abdominal aorta

Twelve of the 21 scans were indicated as a follow-up examination after TEVAR for evaluation of the graft position or endoleakage. Nine patients were scanned due to an aortic aneurysm without any previous intervention. 15 scans included the thorax, abdomen and pelvis region, three scans the thorax only and three scans the abdomen and pelvis region. In 9 patients a previous scan of the same region using iodine-based contrast agent was available for comparison. All 21 cases showed a successful contrast-phase timing and all images were free from relevant motion artifacts. All Gd-angiographies were independently considered to be diagnostic by two different cardiovascular radiologists, each with more than 7 years of experience in reading cardiovascular cases. No disagreements on diagnostic quality occurred. Exemplary images of conventional and MonoE40 reconstructions are shown in Fig. 1.

### Comparison of pre- and post-contrast images

**Fig. 2:**
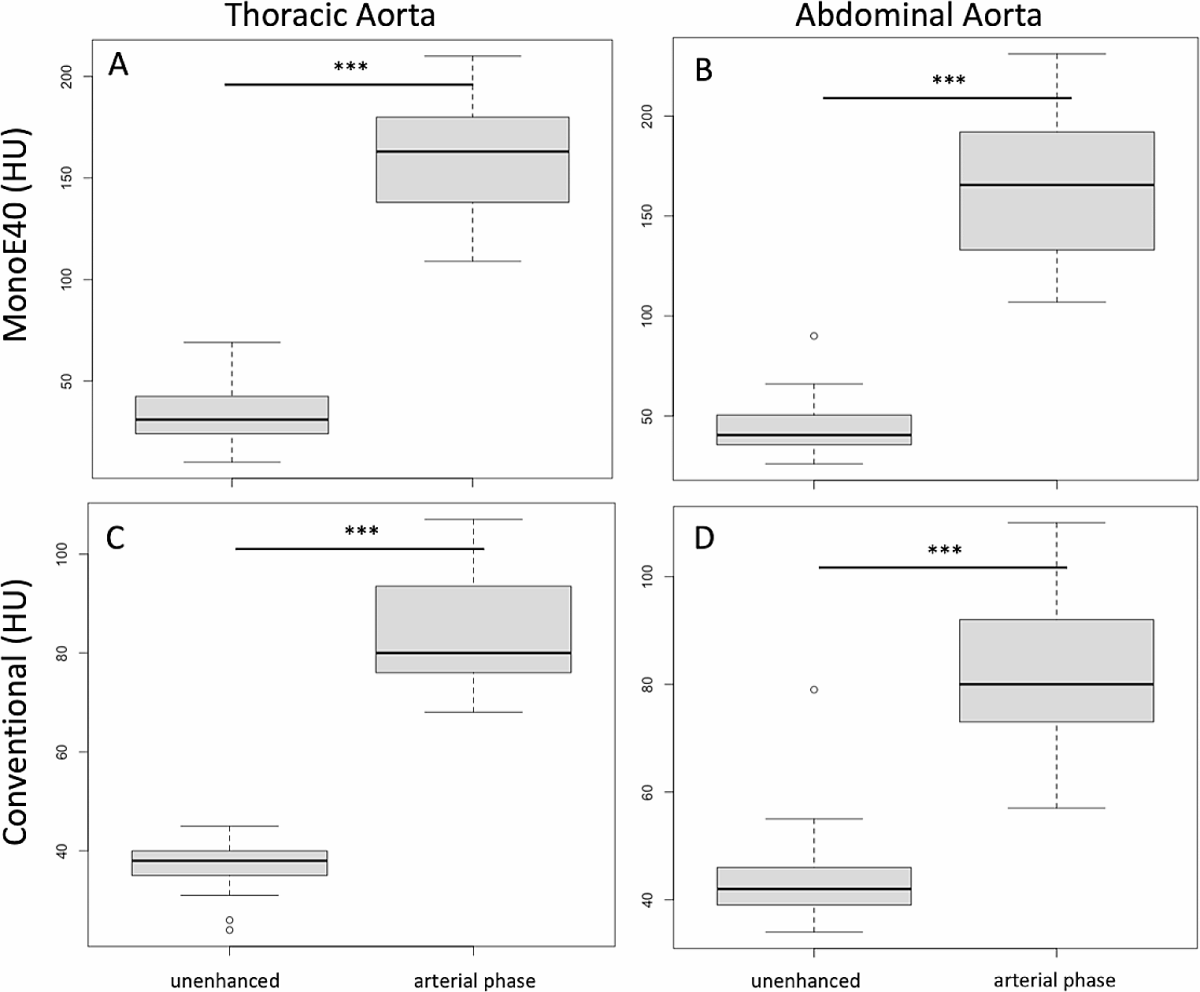
Values of MonoE40 (A, B) and conventional reconstruction (C, D) of the thoracic (A, C) and abdominal Aorta (B, D) on unenhanced images and images after contrast agent administration in the arterial phase. ***: *p* < 0.001. HU = Hounsfield units

There was a significant increase in the intravascular attenuation of the aorta between pre- and post-contrast images for the MonoE40 images from 33.7 ± 15.6 to 162.3 ± 29.9 Hounsfield units (HU) in the thoracic aorta and from 45.1 ± 16.9 to 164.0 ± 35.9 HU in the abdominal aorta (*p* < 0.001 for both) as shown in Fig. 2.

The conventional images also showed a significant increase in attenuation between pre- and post-contrast images from 36.6 ± 5.8 to 84.4 ± 11.4 in the thoracic aorta and from 44.8 ± 10.9 to 81.6 ± 13.3 HU in the abdominal aorta (*p* < 0.001 for both).

### Comparison of attenuation increase between conventional and MonoE40 images

**Fig. 3:**
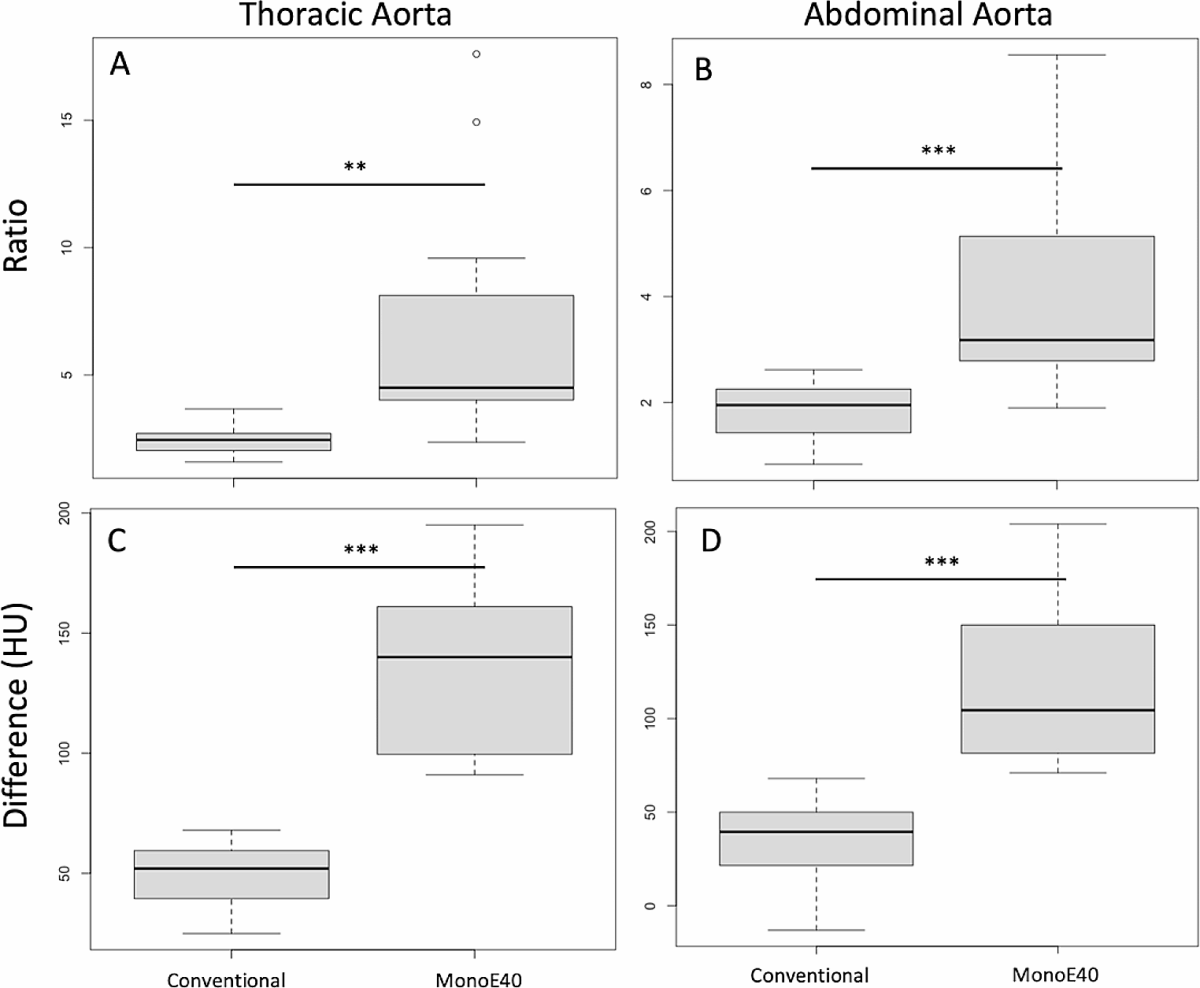
Ratio (A, B) and difference (C, D) in attenuation of the conventional and the MonoE40 reconstructions in the thoracic (A, C) and abdominal Aorta (B, D). **: *p* < 0.01; ***: *p* < 0.001. HU = Hounsfield units

The ratio between pre- and post-contrast images was significantly higher in the MonoE40 images as compared to the conventional images with a factor of 2.4 ± 0.5 vs. 6.5 ± 4.5 in the thoracic aorta (*p* = 0.003) and 1.9 ± 0.5 vs. 4.1 ± 1.8 in the abdominal aorta (*p* < 0.001) as shown in Fig. 3.

**Fig. 4:**
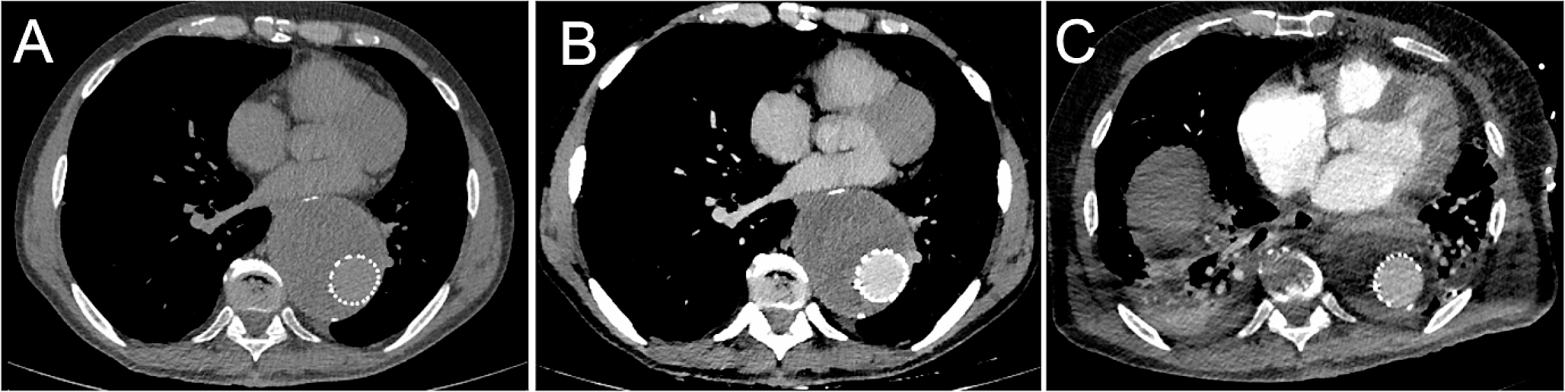
A shows a conventional reconstruction of a Gd-enhanced CTA (arterial phase); mean attenuation of the aorta is 73 HU. In B MonoE40 reconstruction of Gd-enhanced CTA demonstrates the aorta in arterial phase, mean attenuation of the aorta is 191 HU. C illustrates an iodine-enhanced CTA (arterial phase) of the same patient; mean attenuation of the aorta is 153HU.

The absolute difference in attenuation between pre- and post-contrast images was also significantly higher in the MonoE40 images as compared to the conventional images with 49.5 ± 13.3 vs. 134.6 ± 34.0 HU in the thoracic aorta and 39.1 ± 20.0 vs. 116.8 ± 40.7 HU in the abdominal aorta (*p* < 0.001 for both). Figure 4 shows a conventional and MonoE40 reconstruction of a Gd-enhanced CTA (arterial phase) comparing it to an iodine-enhanced CTA (arterial phase) of the same patient.

**Fig. 5:**
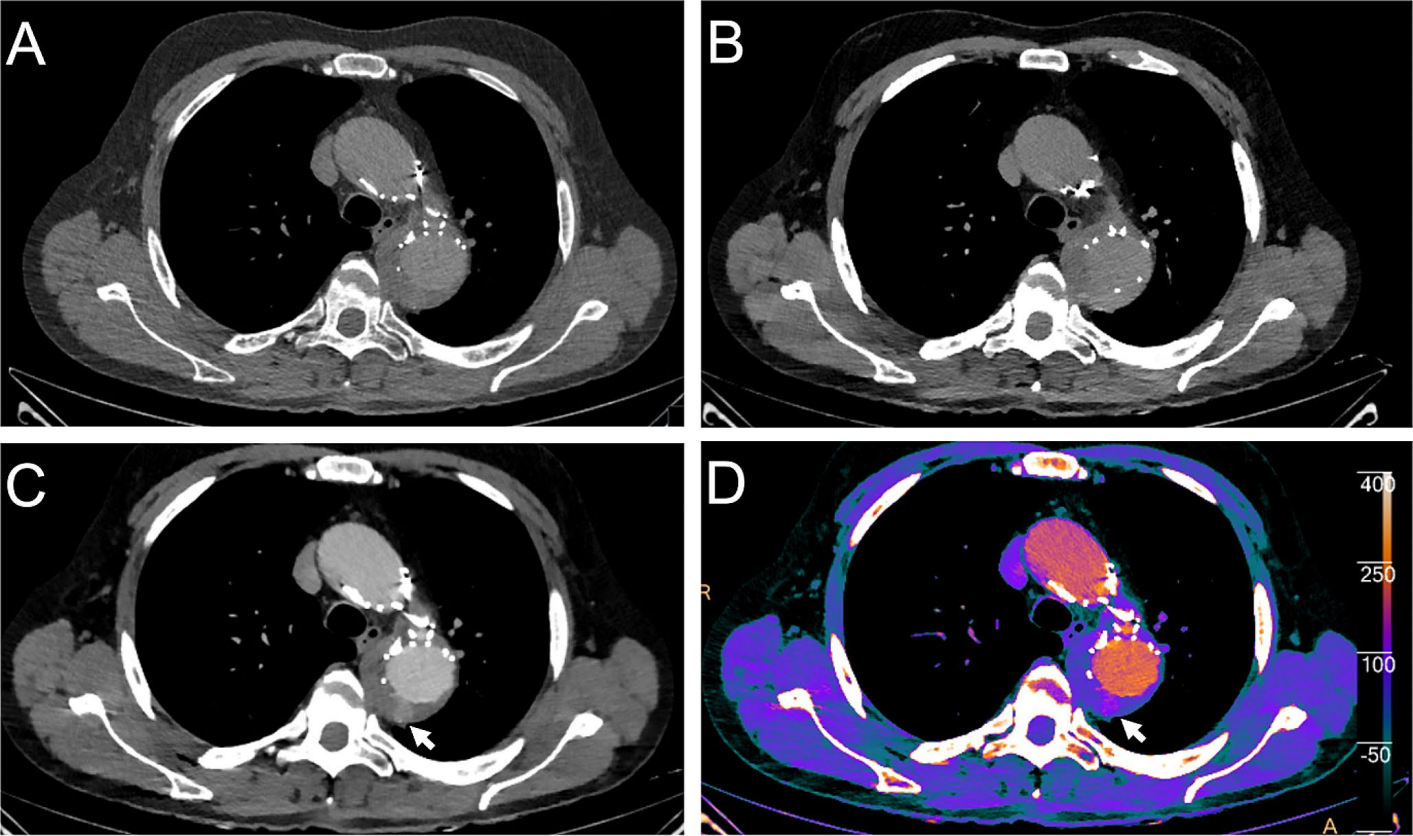
In this figure for image A-C windowing was identical (centre: 100 HU; widths: 600 HU). A shows a conventional reconstruction of a Gd-enhanced CTA (arterial phase); mean attenuation of the aorta is 95 HU. In B MonoE40 reconstruction of the aorta is demonstrated in a venous phase; mean attenuation of the aorta is 80 HU. C illustrates MonoE40 reconstruction of a Gd-enhanced CTA (arterial phase); mean attenuation of the aorta is 220 HU; the white arrow marks the early endoleak. In D a fusion of an arterial MonoE40 with a contrast dye map is shown; the white arrow marks the early endoleak [Note: The contrast dye map is an algorithm developed to highlight iodine. However, Gd can be isolated to a certain extent, too]

Figure 5 shows example images of a post-procedural endoleak which is clearly visible in MonoE40 reconstructions and specific iodine maps, but cannot be discovered on the conventional images.

## Discussion

This is the first study evaluating Gd-CTA utilizing dual energy for the specific diagnostic purpose of aortic assessment for treatment indications. The main findings are (i) the here proposed technique is feasible for a specific application namely diagnostic imaging of the aorta; (ii) MonoE40 reconstructions allow for a relevant and significant increase of attenuation and reach diagnostically recommended thresholds in the aorta; and (iii) Gd-CTA with dual layer CT allows for diagnostic results with high reliability.

Especially in an aging population the number of patients with contraindications to iodine based contrast agents is constantly increasing. High amounts of contrast agents during the EVAR procedure often decrease kidney function limiting the use of CTA for follow examinations [4]. As compared to other theoretical alternatives to iodine based contrast agents such as gold, gadolinium containing contrast agent seems to be a more suitable alternative, due to its broad experience and favorable safety profile in human use, including a lower nephrotoxic potential as well as less allergic reactions reported in literature [15–17]. Follow-up of patients with aortic aneurysms with and without EVAR requires CT scans, which in a routine clinical setting are performed using iodine based contrast agents.

For Gd-based examinations previous studies have shown a low contrast to noise ratio achieved at clinically reasonable contrast agent concentrations when using conventional CT imaging [15, 18]. Despite studies showing feasibility of gadolinium-based CTA in general, very high doses of gadolinium had to be administered to achieve a reasonable contrast and no technique was available to overcome this limitation [8, 18]. Studies by Zhang et al. in rabbits demonstrated a high accuracy for gadolinium-enhanced dual-energy CT pulmonary angiography to detect pulmonary embolism while Gabbai et al. reported high required intravenous doses of gadolinium contrast agent at 1.5 and 2.5 mmol/kg bodyweight [19, 20].

A study by Hamersvelt et al. has shown that dual-layer spectral detector CT (SDCT) allows for overall accurate quantification of gadolinium at both, 120 and 140 kVp in an anthropomorphic thoracic phantom, which is in line with results of this study, acquiring images at 120 kVp [21]. They have proven, that clinically encountered low concentrations of gadolinium, down to 0.5 mg/mL, can be accurately quantified using SDCT under these conditions.

To our knowledge, Nadjiri et al. were the first who were able to achieve a diagnostic attenuation in gadolinium-based CT angiography using MonoE-40 reconstructions at gadolinium concentrations of 0.2 mmol/kg bodyweight in humans [9]. MonoE40 reconstructions reach best results although this method is based on extrapolation of two energy spectra. Otherwise the maximum absorption would be expected at the k-edge of Gd which is 50.2 kEV [22]. However physical characteristics of atoms cannot be simulated by this method. Extrapolation further downwards might even yield better results for the here proposed method.

While the previous publication by Nadjiri et al. comprised several clinical indications including coronary and pulmonary angiography, the present study demonstrates the feasibility of gadolinium based angiography in a well circumscribed clinical application namely CTA of the aorta in patients with aortic aneurysms and follow up after aortic repair. In our clinic the technique has been accepted by clinical partners and is regularly requested for aortic imaging either for follow up after repair or for evaluation. The results from this technique are regarded as sufficient for the purpose by the referring vascular surgeons at our facility.

This study examines SDCT. As alimitation, results might not be transferable on other dual source techniques. While single-source rapid kilovoltage switching might be too slow regarding its offset between the scans dual source dual energy CT also might suffer due to its angular offset. Photon counting CT using dual contrast of iodine and gadolinium based contrast agents or gadolinium based contrast agents alone might become of interest in the future [23, 24].

As a further limitation, our retrospective study evaluates increase in attenuation between conventional and MonoE40 images only and does not evaluate the gold standard of iodine enhanced CTA in a head to head comparison. Future studies in e.g. animal models might consider a direct comparison between these two contrast agents.

Moreover, the use of Gd-based contrast agents in patients with renal failure carries a risk for the development of nephrogenic systemic fibrosis (NSF). According to recent findings, the risk of NSF in patients with Stage IV and V of chronic kidney disease with application of a group II Gd-based contrast agent is 0.07%, suggesting that the diagnostic benefit may often justify the risk [25]. However, each individual case must be evaluated.

To conclude, our study showed that Gd-CTA is a valid and reliable alternative for diagnostic imaging of the aorta for clinical applications. In case of contraindications for iodine-based contrast agent Gd-spectral-CTA should be considered whenever available.

## Data Availability

The data presented in this study are available upon reasonable request from the corresponding author.
